# Sequential Deletions of Interferon Inhibitors MGF110-9L and MGF505-7R Result in Sterile Immunity against the Eurasia Strain of Africa Swine Fever

**DOI:** 10.1128/jvi.01192-22

**Published:** 2022-10-05

**Authors:** Mingyang Ding, Wen Dang, Huanan Liu, Keshan Zhang, Fan Xu, Hong Tian, Huaguo Huang, Zhengwang Shi, Yongjie Sunkang, Xiaodong Qin, Yong Zhang, Haixue Zheng

**Affiliations:** a State Key Laboratory of Veterinary Etiological Biology, College of Veterinary Medicine, Lanzhou University, Lanzhou Veterinary Research Institute, Chinese Academy of Agricultural Sciences, Lanzhou, China; b College of Veterinary Medicine, Gansu Agricultural University, Lanzhou, China; c College of Veterinary Medicine, Northwest A&F University, Yangling, Shaanxi, China; Lerner Research Institute, Cleveland Clinic

**Keywords:** ASFV, MGF110-9L, MGF505-7R, attenuation, protective efficacy

## Abstract

African swine fever virus (ASFV) causes significant morbidity and mortality in pigs worldwide. The lack of vaccines or therapeutic options warrants urgent further investigation. To this aim, we developed a rationally designed live attenuated ASFV-Δ110-9L/505-7R mutant based on the highly pathogenic Genotype II ASFV CN/GS/2018 backbone by deleting 2 well-characterized interferon inhibitors MGF110-9L and MGF505-7R. The mutant was slightly attenuated in vitro compared to parental ASFV but highly tolerant to genetic modifications even after 30 successive passages *in vitro*. Groups of 5 pigs were intramuscularly inoculated with increasing doses of the mutant, ranging from 10^3^ to 10^6^ hemadsorption units (HAD_50_). Thirty-five days later, all groups were challenged with 10^2^ HAD_50_ of virulent parental ASFV. All the animals were clinically normal and devoid of clinical signs consistent with ASFV at the period of inoculation. In the virulent challenge, 2 animals from 10^3^ HAD_50_-inoculated group and 1 animal from 10^4^ HAD_50_-inoculated group were unprotected with severe postmortem and histological lesions. The rest of animals survived and manifested with relatively normal clinical appearance accompanied by tangible histological improvements in the extent of tissue damage. Meanwhile, antibody response, as represented by p30-specific antibody titers was positively correlated to protective efficacy, potentializing its usage as an indicator of protection. Moreover, compared to 1 dose, 2 doses provided additional protection, proving that 2 doses were better than 1 dose. The sufficiency in effectiveness supports the claim that our attenuated mutant may be a viable vaccine option with which to fight ASF.

**IMPORTANCE** African swine fever virus (ASFV) is a causative agent of acute viral hemorrhagic disease of domestic swine which is associated with significant economic losses in the pig industry. The lack of vaccines or treatment options requires urgent further investigation. ASFV MGF110-9L and MGF505-7R, 2 well-characterized interferon inhibitors, were associated with viral virulence, host range, and immune modulation. In this study, a recombinant two-gene deletion ASFV mutant with deletion of MGF110-9L and MGF505-7R was constructed. The result showed that the mutant was safe, and also highly resistant to genetic modification even after 30 successive passages. High doses of our mutant (10^5^ and 10^6^ HAD_50_) provided sterile immunity and complete protection in a virulent challenge. Two doses were superior to 1 dose and provided additional protection. This study develops a new ASFV-specific live attenuated vaccine and may be a viable vaccine option against ASF.

## INTRODUCTION

African swine fever virus (ASFV), the etiological agent of a highly contagious hemorrhagic viral disease of domestic and wild pigs (1), is the only member of the *Asfarviridae* family, genus *Asfivirus* (2, 3). It is characterized with a multi-layered icosahedral morphology composing of an internal core, an internal lipid membrane, an icosahedral capsid, and an outer lipid envelope (4, 5). Based on the C-terminus of B646L gene encoding the p72 protein, ASFV is divided into 24 genotypes, with Genotype II dominantly circulating throughout Europe, the Russian Federation, the People’s Republic of China, and Southeast Asia (6, 7). African swine fever (ASF) outbreaks have caused great economic consequences at the global level. Currently, since no vaccines or therapeutic options are available to prevent the infection, control of the disease solely depends on administrative and regulatory measures derived from classical disease control strategies (8).

ASFV has a linear dsDNA genome structure of approximately 170–194 kbp in size. It contains a relatively conserved, evolutionary stable, and centrally-located core of replication-associated genes flanked by left and right variable regions (LVR and RVR) with more variability in size and gene content (9). Understanding ASFV genetic signatures is critical for uncovering ASFV antigenic and phenotypic diversity that is problematic for developing ASF vaccines. Through exploiting a comparative genomics approach to systematically study 46 genomes of ASFV, it was revealed that ASFV had an open pan-genome encompassing 151–174 genes, among which 86 were identified as core genes and the remainder as flexible accessory genes (10, 11). The latter were predominantly composed of multigene families (MGF) of paralogous genes. Until now, 5 MGFs (MGF100, MGF110, MGF300, MGF360, and MGF505/530) have been defined with variable compliments in different ASFV strains (12). Interestingly, functions of many MGFs are still unknown. Reconstruction of the ancestral states of MGFs along with ASFV phylogeny demonstrated that most MGFs undertook either the copy number variations or the gain-or-loss of changes, which tended to occur within strains of the same genotype (12). Notably, several well-characterized MGFs were found to be implicated in pathogenesis and immune evasion (13–18).

Noticeable is the fact that protective immunity against ASF is achievable by hypovirulent ASFV strains, prompting the development of live attenuated ASFV vaccines, either naturally isolated or obtained by genetic manipulation. Deliberate deletion of a subset of non-essential genes, including I177L, TK (A240L), UK (DP96R), 9GL, NL (DP71L), CD2v and DP148R led to attenuation to some extent in different ASFV isolates. Deletion of the viral CD2v (EP402R) gene from the virulent BA71 strain of genotype I attenuated the strain and offered cross-protective capability against lethal challenge with Georgia 2007/1, the genotype II strain of ASFV circulating in continental Europe (19). Notably, 9GL was found to confound virion maturation and viral growth *in vitro*. Deleting 9GL exerted differential effects, resulting in complete attenuation in Malawi Lil-20/1 (MAL) but partial attenuation in Georgia 2007 (ASFV-G). As anticipated, both 9GL-deficient mutants induced an effective protection against homologous challenge (20, 21). The I177L gene was recently identified as a new virulence-associated gene, as deletion of the I177L gene in ASFV-G resulted in sterile immunity against homologous challenge, irrespective of administration route (22–24).

Complete attenuation is not easily achieved by simply deleting one single virulence-associated gene in ASFV. Meanwhile, the stability of one-gene-mediated genetic modification raises safety concerns that need to be addressed, as ASFV mutants tended to convert to the virulent strain by acquiring the lost gene through the recombination events, the most frequently identified drivers of double-stranded DNA viruse’s evolution. Consequently, some advances have been made by combinational deletions of multiple virulence-associated genes. Simultaneous deletion of the 9GL and UK genes in ASFV Georgia 2007 isolate offered increased safety and protection against homologous challenge. Protection from disease was achieved as early as 2 weeks after vaccination, even when the pigs were exposed to a higher-than-normal concentration of highly virulent ASFV. Meanwhile, 2 deletions in 2 separate areas of the virus substantially decreased the potential of genotype and phenotype reversion, resulting in boosted safety (25). Moreover, deletion of MGF110-9L in ASFV CN/GS/2018 isolate and deletion of MGF505-7R in ASFV HLJ/18 strain, both belonging to the currently circulating genotype II viruses in China, resulted in pronounced but not complete attenuation of the viruses in swine (13, 16). Aiming to enhance attenuation and increase safety, we reported the construction of a double-gene-deleted recombinant ASFV-Δ110-9L/505-7R mutant. Intramuscular inoculation of pigs with increasing doses of ASFV-Δ110-9L/Δ505-7R mutant, ranging from 10^3^ to 10^6^ hemadsorption units (HAD_50_), are safe without severe side effect. In the virulent challenge, low doses of 10^3^ and 10^4^ HAD_50_ provided clinically significant 60% and 80% protection, respectively, whereas high doses of 10^5^ and 10^6^ HAD_50_ protected 100% of pigs from fatal disease. Meanwhile, assessment of antibody response demonstrated that p30-specific antibody is positively correlated with protective efficacy, potentializing its usage as a potential indicator for protection. Moreover, compared to 1 dose inoculation procedure, 2 doses provided additional protection. Our results rationalized the understanding of ASFV gene function, virus attenuation, and protection against infection.

## RESULTS

### The recombinant ASFV-Δ110-9L/505-7R mutant displayed replication defect relative to parental ASFV.

**FIG 1:**
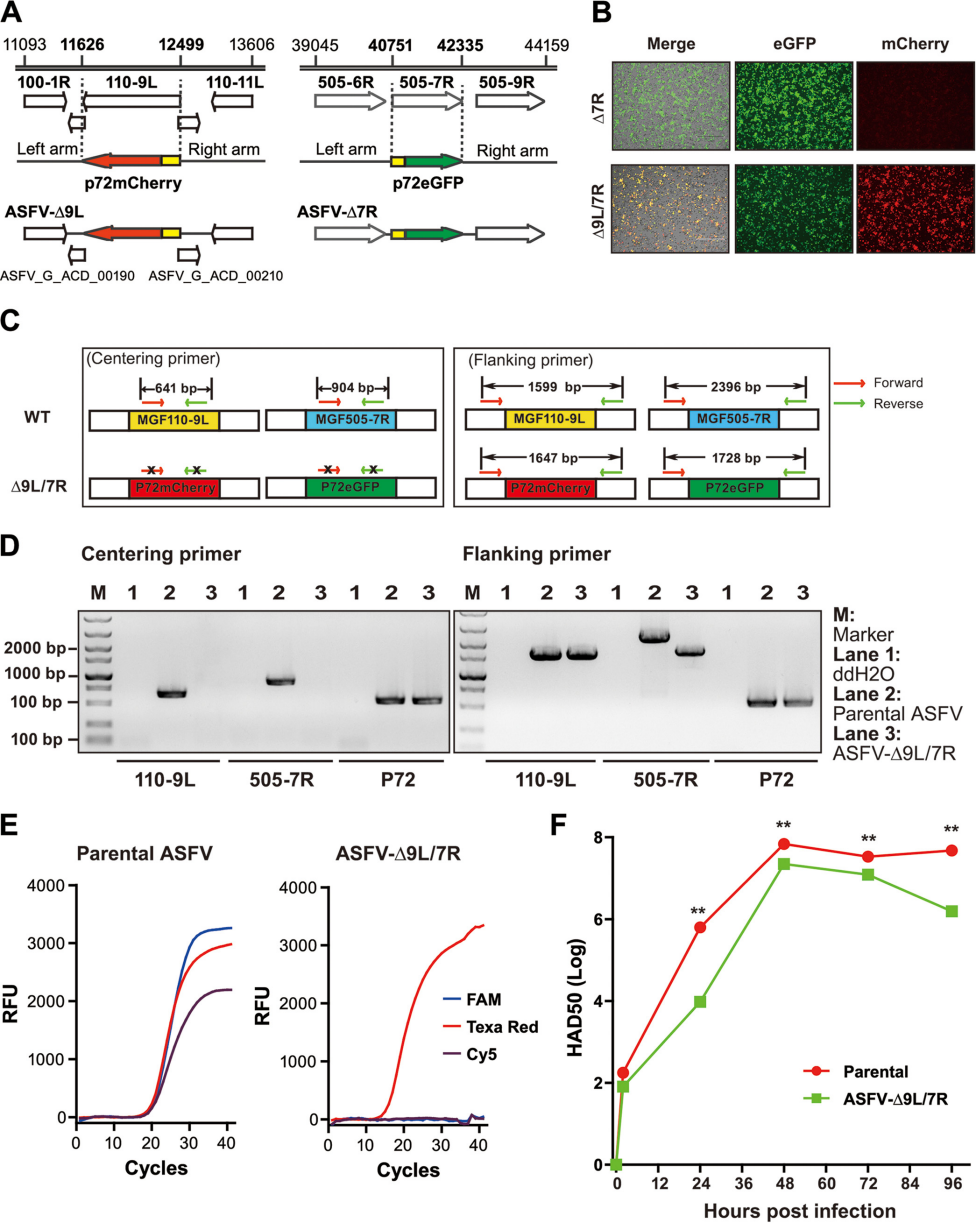
Construction and characterization of the two-gene-deleted ASFV-Δ110-9L/505-7R mutant. (A) Schematic demonstration of constructing the ASFV-Δ110-9L/505-7R mutant. Two transfer vectors, namely, pASFV-Δ9L and pASFV-Δ7R, were constructed in the pUC57 backbone by replacing MGF110-9L and MGF505-7R with reporter gene cassettes p72mCherry and p72eGFP, respectively. Nucleotide positions indicative of the boundaries of deletion and replacement relative to parental ASFV CN/GS/2018 genome were labeled. (B) Fluorescent microscopy of BMDM after 48 h infection with the intermittent ASFV-Δ505-7R mutant (upper) and the ASFV-Δ110-9L/505-7R mutant at 0.1 MOI. (C) Schematic of designing 2 pairs of primers located within (centering primer) and outside (flanking primer) of the deleted genes. The expected sizes of PCR products were indicated. (D) Assessment of genetic deletions and replacements by PCR analysis. Viral DNA obtained from parental ASFV or ASFV-Δ110-9L/505-7R mutant were subjected to PCR amplification. In parallel, a pair of primers targeting viral p72 gene was included as an indicator of genome input. (E) Development of triplex rPCR assay for detection and differentiation of ASFV-Δ110-9L/505-7R mutant and parental ASFV. Compared to parental ASFV, amplification curves of ASFV-Δ110-9L/505-7R mutant displayed positive signals for viral p72 but not MGF110-9L nor MGF505-7R, implying the combinational deletions of targeted genes. (F) Kinetic replication curves (mean) of ASFV-Δ110-9L/505-7R mutant and parental ASFV following infection of BMDM at 0.1 MOI.

Combinational deletions of virulence-associated genes are promising routes for construction of rationally attenuated ASFV candidate vaccine strains. Here, we constructed an ASFV recombinant mutant, namely, ASFV-Δ110-9L/505-7R with double deletions of MGF110-9L and MGF505-7R as well as interruption of uncharacterized genes ASFV_G_ACD_00190 and ASFV_G_ACD_00210 in the genome of the highly virulent ASFV CN/GS/2018 strain currently circulating in China (Fig. 1A). Correspondingly, MGF110-9L and MGF505-7R were replaced with p72mCherry and p72eGFP reporter gene cassettes, respectively, generating the recombinant ASFV-Δ110-9L/505-7R mutant simultaneously positive for eGFP and mCherry signals (Fig. 1B). The identity of the mutant was confirmed by PCR using ASFV viral DNA and primers specifically binding outside or inside of the target genes (Fig. 1C). As anticipated, no bands appeared after PCR amplification with both centering primer pairs in the mutant, suggestive of simultaneous deletions of MGF110-9L and MGF505-7R genes (Fig. 1D; left panel). Differential PCR products corresponding to the expected molecular sizes were amplified using viral DNA extracted from parental ASFV and the mutant by both flanking primer pairs, primarily indicative of successful insertions of the reporter gene cassettes (Fig. 1D; right panel). The precision of genetic modifications was further confirmed by sequencing of the amplified PCR products (Fig. S1 and Fig. S2). In this study, a triplex real-time PCR assay was developed for detection and differentiation of the mutant and parental ASFV. Consistently, parental ASFV DNA samples showed positive amplification curves for the 3 fluorescence channels (Fig. 1E; left panel). However, the results with DNA samples obtained from the mutant showed only Texa Red fluorescence curve by the triplex rPCR assay (p72 signal), further confirming simultaneous deletions of MGF110-9L and MGF505-7R (Fig. 1E; right panel).

To assess the precision of designed genetic modifications and the emergence of unwanted genetic modifications outside the deleted genes, next-generation sequencing of the genome obtained from ASFV-Δ110-9L/505-7R was also performed. Our next-generation sequencing (NGS) results demonstrated the accuracy of the designed genetic modifications, as MGF110-9L ORF and MGF505-7R ORF being precisely replaced by the p72mCherryΔMGF110-9L reporter cassette and p72eGFPΔMGF505-7R reporter cassette, respectively. Those results were aligned with the Sanger sequencing data obtained from traditional PCR using flanking primers. Besides the designed genetic modifications, our NGS results demonstrated the following unwanted additional mutations in Table 1: four nucleotide deletions (a CC, a G, a GG, and an A at nucleotide positions 26, 16475, 20427, and 175270, respectively) and a nucleotide insertion (an A at nucleotide position 16260) in non-coding regions (NCR); two silent mutations at nucleotide position 117734 in ORF CP2475L and 146425 in ORF S237R; four residue substitutions, namely, Val to Ala at residue position 50 in ORF A104R, Asp to Gly at residue position 455 and 1062 in ORF M1249L, and Val to Leu at residue position 70 in ORF D205R. How those unwanted additional mutations affect the phenotype of the mutant await further investigation.

**TABLE 1:** Summary of differences between the full-length genome sequence of ASFV-Δ110-9L/505-7R and the parental ASFV CN/GS/2018

NPN^*a*^		Region or ORF, description of modification^*b*^	
26		NCR, deletion of CC	
16260		NCR, insertion of G	
16475		NCR, deletion of G	
20427		NCR, deletion of GG	
47093		A104R, T to C (Val50Ala)	
75867		M1249L, T to C (Asp1062Gly)	
77688		M1249L, T to C (Asp455Gly)	
117734		CP2475L, A to G (Gly2190Gly), SM	
137313		D205R, G to C (Val70Leu)	
146425		S237R, T to C (Gly44Gly), SM	
175270		NCR, deletion of A	

a NPN, nucleotide position number based on the sequence of parental strain ASFV CN/GS/2018.

b NCR, noncoding region; SM, the nucleotide modification caused a silent mutation.

Evaluation of the growth kinetics revealed that the mutant replicated productively, with the growth curve being similar to that of parental ASFV. Notably, replication of the mutant was delayed with the yield being significantly lower at any time points studied (Fig. 1F). Collectively, ASFV-Δ110-9L/505-7R harboring simultaneous deletion of 2 virulence-associated genes displayed decreased replication capability relative to parental ASFV.

### The mutant is highly tolerant to genetic modifications following 30 serial passages *in vitro*.

**FIG 2:**
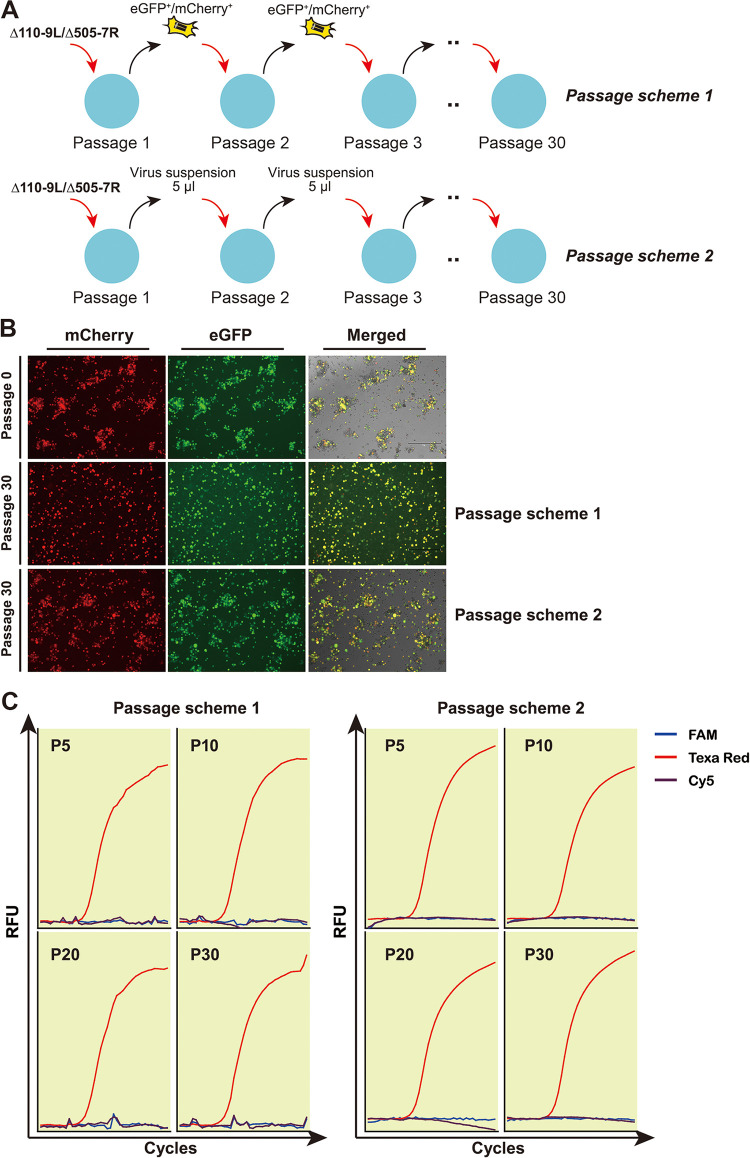
The ASFV-Δ110-9L/505-7R mutant is highly tolerant to genetic modifications following 30 successive passages. (A) Schematic representation of passage schemes designed for ASFV-Δ110-9L/505-7R mutant. A total of 30 successive passages *in vitro* were performed. (B) Fluorescent microscopy of cells infected with the initial ASFV-Δ110-9L/505-7R mutant and subsequent viruses at passage 30. (C) Assessment of the stability of simultaneous deletions in the genome of the mutant at passages 5 (P5), 10 (P10), 20 (P20) and 30 (P30) by using triplex rPCR.

Genetic deletions conferred the mutant minor attenuation of *in vitro* replication. However, whether combinational exclusion of MGF110-9L and MGF505-7R in the mutant was restored during serial passages *in vitro* was not answered, as recombination is one of the most frequently identified drivers of double-stranded DNA virus evolution (26). For this purpose, the mutant was serially passaged in MBDM for 30 times, approximately 72 h/passage (Fig. 2A). Irrespective of passage schemes, the mutant was highly tolerant to fluorescent reporter gene cassettes, as evidenced by high fluorescent intensities of eGFP/mCherry signals inside virus-infected cells (Fig. 2B). Similarly, our triplex rPCR assay amplified the target sequence of p72 but not MGF110-9L nor MGF505-7R in the genome of the mutant obtained at passages 5 (P5), 10 (P10), 20 (P20) and 30 (P30) (Fig. 2C). Those data collectively demonstrated that the mutant was highly tolerant to foreign-gene insertion, potentializing its usage as a vaccine candidate.

### The mutant displays safety even at a high dose of 10^6^ HAD_50_.

**FIG 3:**
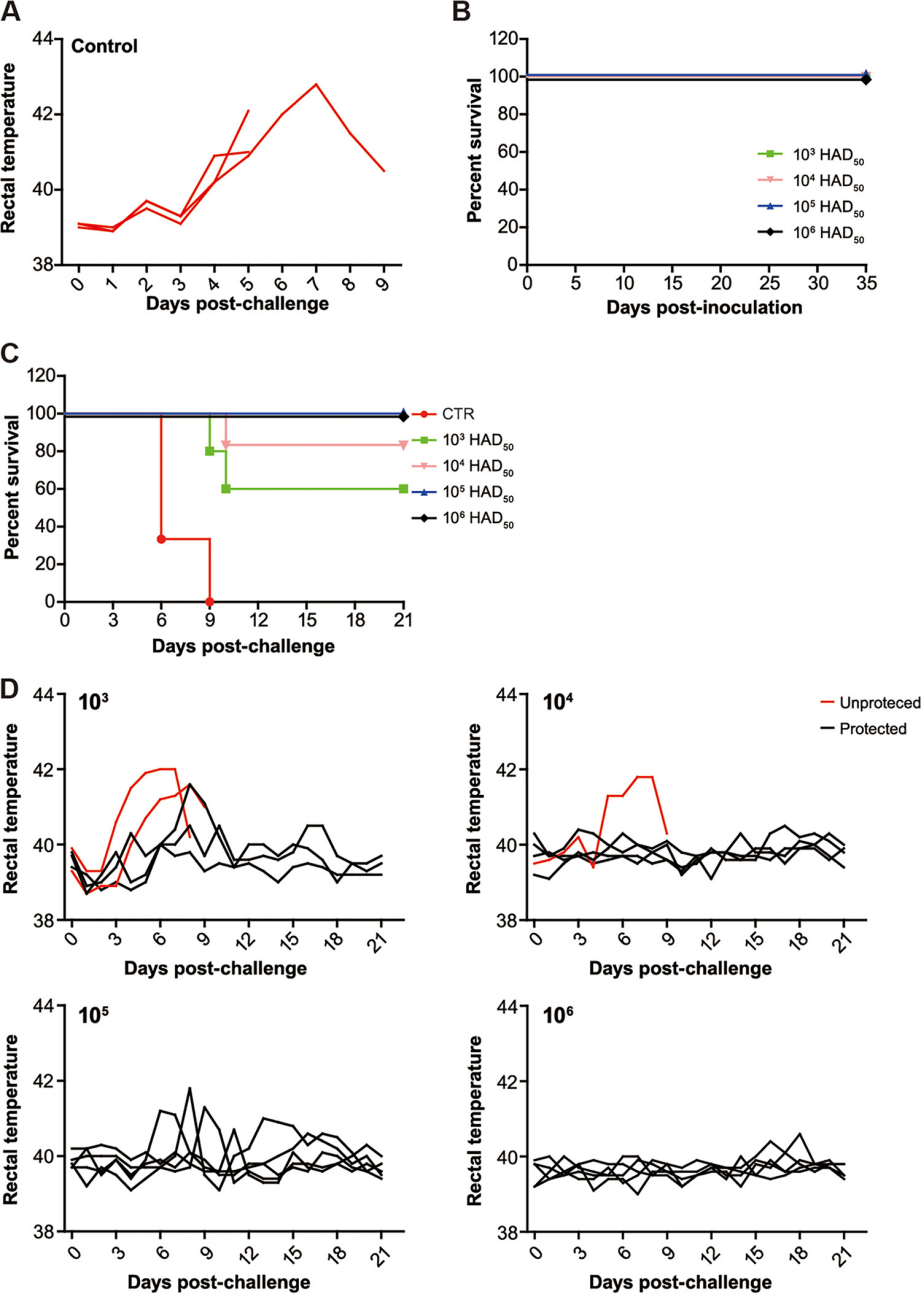
Descriptive survival analyses and rectal temperatures of experimental pigs. The animals were primarily inoculated IM with the ASFV-Δ110-9L/505-7R mutant at each indicated doses. At 35 days postinoculation, animals were subsequently exposed to a virulent challenge with an extension of the monitoring period to 21 days. Meanwhile, group of three pigs receiving a lethal dose of parental ASFV was included. (A) Rectal temperatures of three pigs from control group following infection with virulent ASFV. (B) Survival rate of pigs at the period of inoculation. (C) Survival rate of pigs in the virulent challenge. (D) Rectal temperatures of pigs in the virulent challenge.

To assess the potential use of the mutant as live attenuated vaccine candidate, we evaluated its virulence and protective efficacy *in vivo* using our well-established pig model of ASFV infection. To that end, five 4-week-old piglets were inoculated intramuscular (IM) with increasing doses of the mutant. Simultaneously, 3 animals received 10^2^ HAD_50_ of parental ASFV CN/GS/2018, a lethal dose expected to cause 100% death in piglets, as previous study showed that pigs inoculated with as low as 10 HAD_50_ developed severe ASFV-related clinical symptoms and died within 15 days (15). As anticipated, all 3 animals succumbed to parental ASFV at day 6, 6 and 9 after inoculation coupled with severe ASFV-specific side effect, in particular, acute high fever (40.5-42°C) (Fig. 3A and Table 2). Importantly, all piglets survived the mutant inoculation, irrespective of injection dosages (Fig. 3B and Table 2). Specifically, piglets inoculated with low doses of the mutant, namely, 10^3^ and 10^4^ HAD_50_, stayed free of any ASFV-related clinical symptoms (Table 2). Only one animal from each group receiving high doses of 10^5^ and 10^6^ HAD_50_ demonstrated short-term recurrent fever at the very beginning of inoculation. Collectively, high doses of the mutant are safe in pigs, prompting evaluation of protection against a virulent challenge.

**TABLE 2:** Summary of swine survival and rectal temperatures at the period of inoculation^*a*^

Group	Dose (HAD_50_)	No. of survivors	Time to death (mean)(days)^*b*^	Summary of fever response		
				No. of pigs with fever/total^*c*^	Duration (days)	Maximum daily temp (°C)
Control	10^2^ (parental)	0/3	7	3/3	3.3	42.8
ASFV-Δ110-9L/505-7R	10^3^	5/5		0/5	0	40.1
	10^4^	5/5		0/5	0	40.4
	10^5^	5/5		1/5	5	40.5
	10^6^	5/5		1/5	5	40.6

a A group of three pigs receiving a lethal dose of parental ASFV was also included.

b All animals were euthanized due to the welfare regulations according to the corresponding IACUC protocol.

c Pigs with a rectal temperature over 40°C for three consecutive days or above 40.5°C were defined as fever.

### High doses of the mutant confer protection against a virulent challenge.

After a virulent challenge with a lethal dose of 10^2^ HAD_50_ of parental ASFV CN/GS/2018, low doses only confer partial protection, as 2 animals from 10^3^ HAD_50_-inoculated group and 1 animal from 10^4^ HAD_50_-inoculated group developed ASF-compatible clinical signs and died, even though a slight delay in death was observed when compared to the parental ASFV-inoculated animals (average time to death, 9.5 day vs 10 day vs 7 day) (Fig. 3C and Table 3). Apart from that, several animals from 2 groups suffered from continuous high fever but survived from ASFV infection. Surprisingly, high doses of 10^5^ and 10^6^ HAD_50_ provided complete protection against parental ASFV attack. Noticeable is the fact that 2 animals receiving 10^5^ HAD_50_-inoculation manifested with transient and fluctuating fever, but animals from 10^6^ HAD_50_-inoculation group were asymptomatic and appeared clinically healthy (Fig. 3D and Table 3).

**TABLE 3:** Summary of swine survival and rectal temperatures in the virulent challenge^*a*^

Group	Dose (HAD_50_)	No. of survivors	Time to death (mean)(days)^*b*^	Summary of fever response		
				No. of pigs with fever/total^*c*^	Duration (days)	Maximum daily temp (°C)
Control	NA^*d*^	0/3	7	3/3	3.3	42.8
ASFV-Δ110-9L/505-7R	10^3^	3/5	9.5	3/5	5.5	42
	10^4^	5/6	10	2/6	5	41.8
	10^5^	5/5		2/5	2.5	41.1
	10^6^	5/5		0/5	0	40.4

a A group of three pigs receiving a lethal dose of parental ASFV was also included.

b All animals were euthanized due to the welfare regulations according to the corresponding IACUC protocol.

c Pigs with a rectal temperature over 40°C for three consecutive days or above 40.5°C were defined as fever.

d Pigs in the control group received no ASFV-Δ110-9L/505-7R before the virulent challenge.

### Animals surviving from the virulent challenge are devoid of viremia.

**FIG 4:**
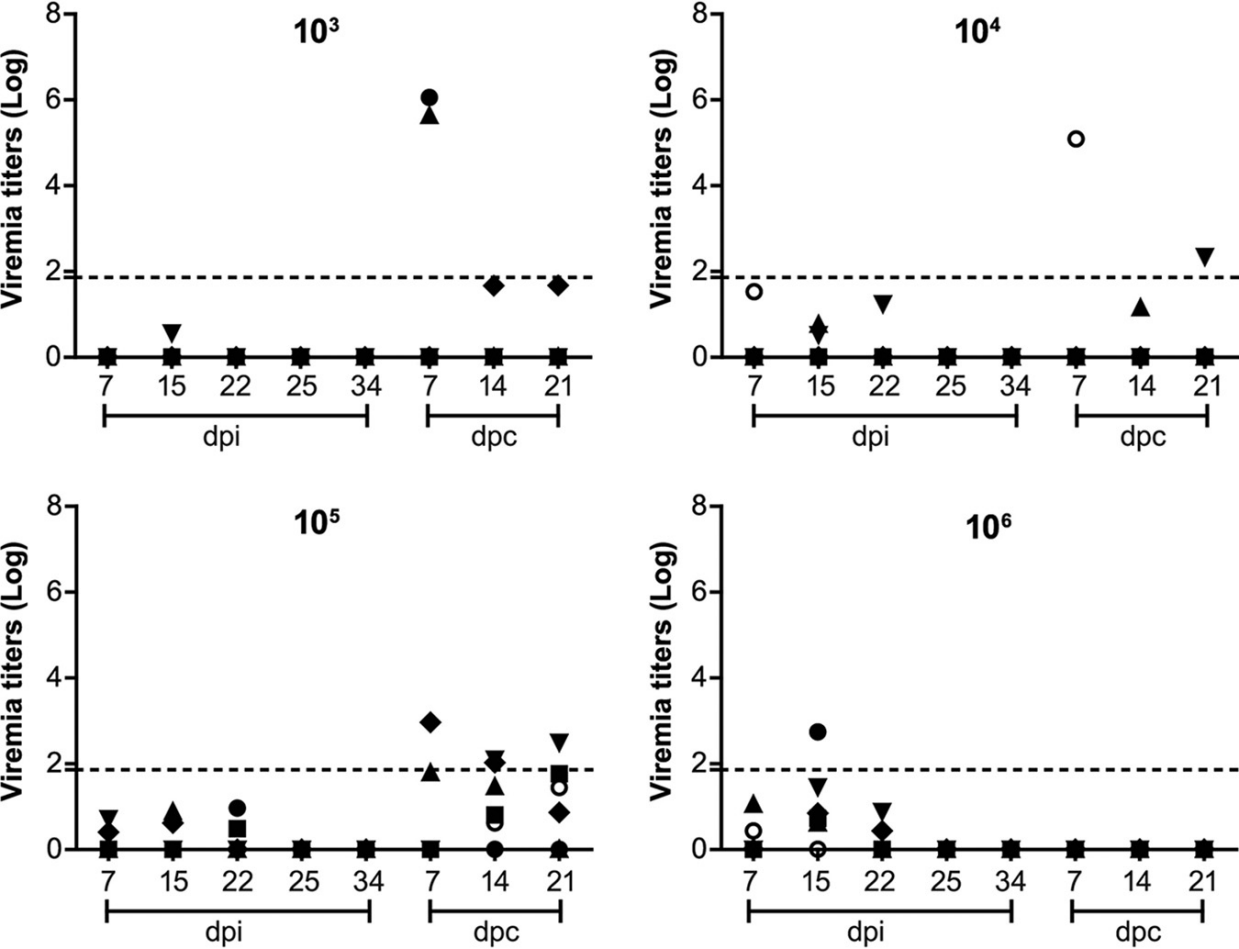
Viremia of experimental pigs. Groups of five pigs inoculated with increasing titers of ASFV-Δ110-9L/505-7R were challenged i.m. with lethal wild type ASFV. Virus DNA was monitored in the blood collected from pigs during the inoculation period and the challenge period.

As predicted, unnoticeable viremia was developed as early as 7 days among inoculated pigs, with extremely low ASFV DNA load being sporadically detected in the serum samples, irrespective of injection dosages, suggestive of *in vivo* safety properties of the mutant (Fig. 4A). Surprisingly, monitoring of serum samples postchallenge demonstrated that totally-protected animals are incapable of developing serious viremia, though mild and harmless viremia were transiently and infrequently detected in several animals. As anticipated, serum from unprotected animals were positive for high-level of viremia, approximately over 10^5^ copies per 1 mL. Consistently, inoculation with the mutant protected host against fecal and oro-nasal viral shedding, apart from the fact that several swabs are doubtfully and transiently positive for ASFV DNA (data not shown). These findings indicated that the mutant had a substantially attenuated phenotype *in vivo* while retaining the capability to induce a protective immune response against a subsequent homologous lethal challenge.

### Totally-protected pigs are free of noticeable ASFV-related postmortem lesions.

**FIG 5:**
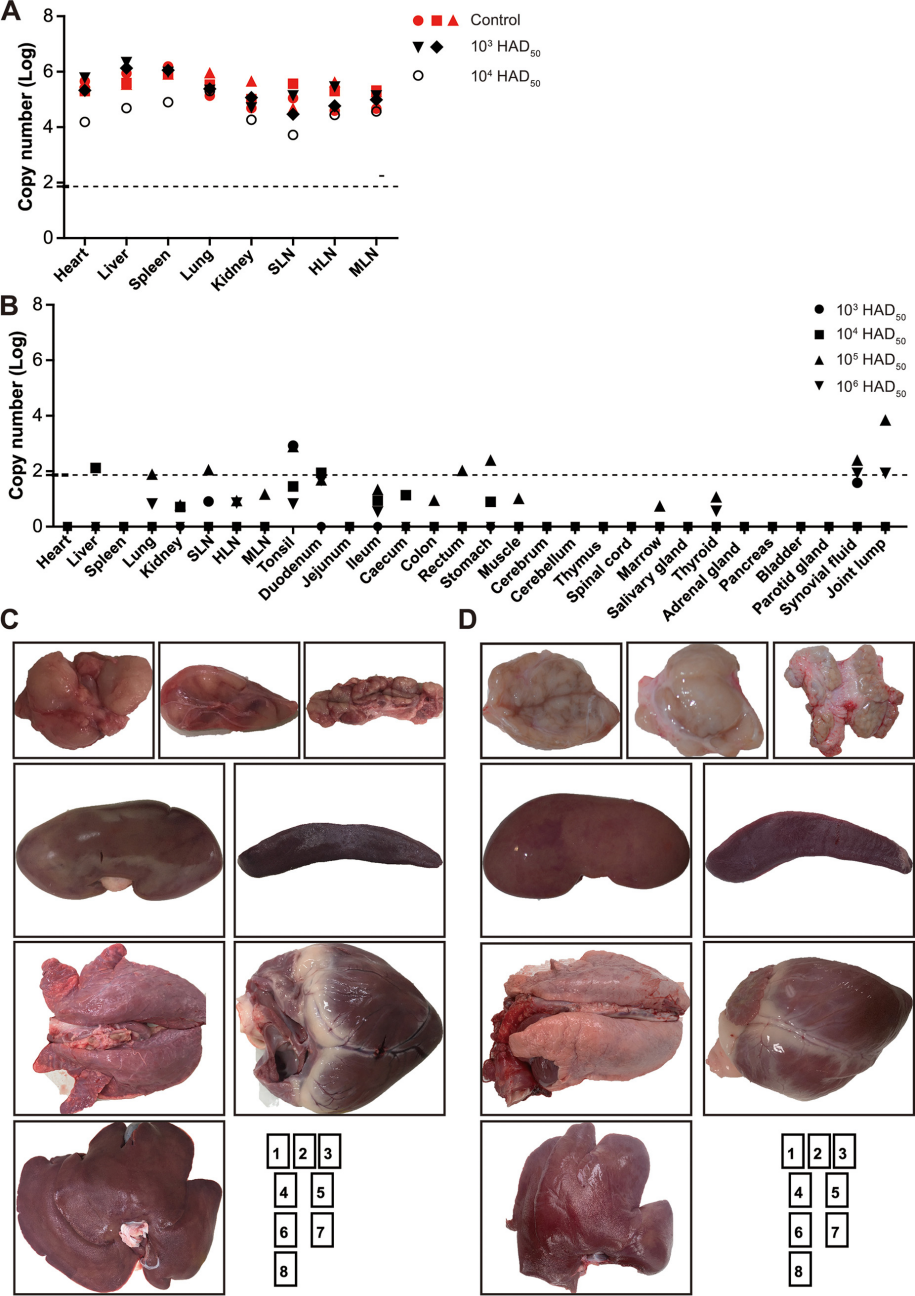
High viral loads correlated significantly with tissue abnormalities. (A) The DNA was extracted from tissue samples and subjected to quantification of ASFV genome copy number. Viral loads of tissue samples obtained from unprotected pigs resembled the results from control pigs. The dashed lines indicate the lower limit of detection. (B) Viral loads of tissue samples from totally-protected pigs were significantly diminished. One surviving pig from each of the groups was examined and representatively displayed. SLN, submandibular lymph node; HLN, gastrohepatic lymph node; MLN, mesenteric lymph node. (C and D) Representative figures for tissue pathology in one unprotected and one totally-protected pigs collectively from 10^3^ HAD_50_-inoculated group as follows: 1, SLN; 2, HLN; 3, MLN; 4, kidney; 5, spleen; 6, lung; 7, heart; and 8, liver.

The severity and distribution of the lesions caused by ASFV were tightly correlated with viral loads. On average, in the context of control pigs infected with parental ASFV, the highest levels (> 10^6^ copies/μg DNA) of viral DNA were detected in spleen and liver. Besides, comparatively high levels of viral DNA, ranging from 10^5^ to 10^6^ copies/μg of DNA, were observed in the remaining tissue samples. Significantly, viral loads in the tissue samples from unprotected animals were indistinguishable from control animals, albeit the fact that 10^4^ HAD_50_ inoculation seemingly slightly decreased viral loads in several tissue samples obtained from an unprotected animal (Fig. 5A). However, viral loads in the totally-protected animals were greatly altered by approximately 2–4 log decrease, reaching Limit of Detection of about 10^1.86^ copies/μg DNA (Fig. 5B).

The inferences were further validated by a combined measure of postmortem lesions. Systemic and gross changes, in particular enlarged and hemorrhagic lymph nodes accompanied with inflammation, were observed in all ASFV-infected control pigs and unprotected pigs, as representatively demonstrated by a euthanized 10^3^ HAD_50_-inoculated pig (Fig. 5C). Moreover, renal lesions varying from slight cortical petechia to renomegaly with diffuse hemorrhage and congestion were evident (Fig. 5C). As expected, in the case of totally-protected animals, external lesions were enormously relieved as proved by the findings that the lymph nodes were externally white/pink in color, coupled with uniformly colored and textured tissue samples examined, as represented by a surviving pig from 10^3^ HAD_50_-inoculated group in Fig. 5D, and 1 animal from each group of 10^4^, 10^5^, and 10^6^ HAD_50_-inoculated group in Fig. S3. Those findings provide more details about the correlation between ASFV replication and the degree of tissue involvement and resulting tissue damage.

### The mutant protects against tissue pathology.

**FIG 6:**
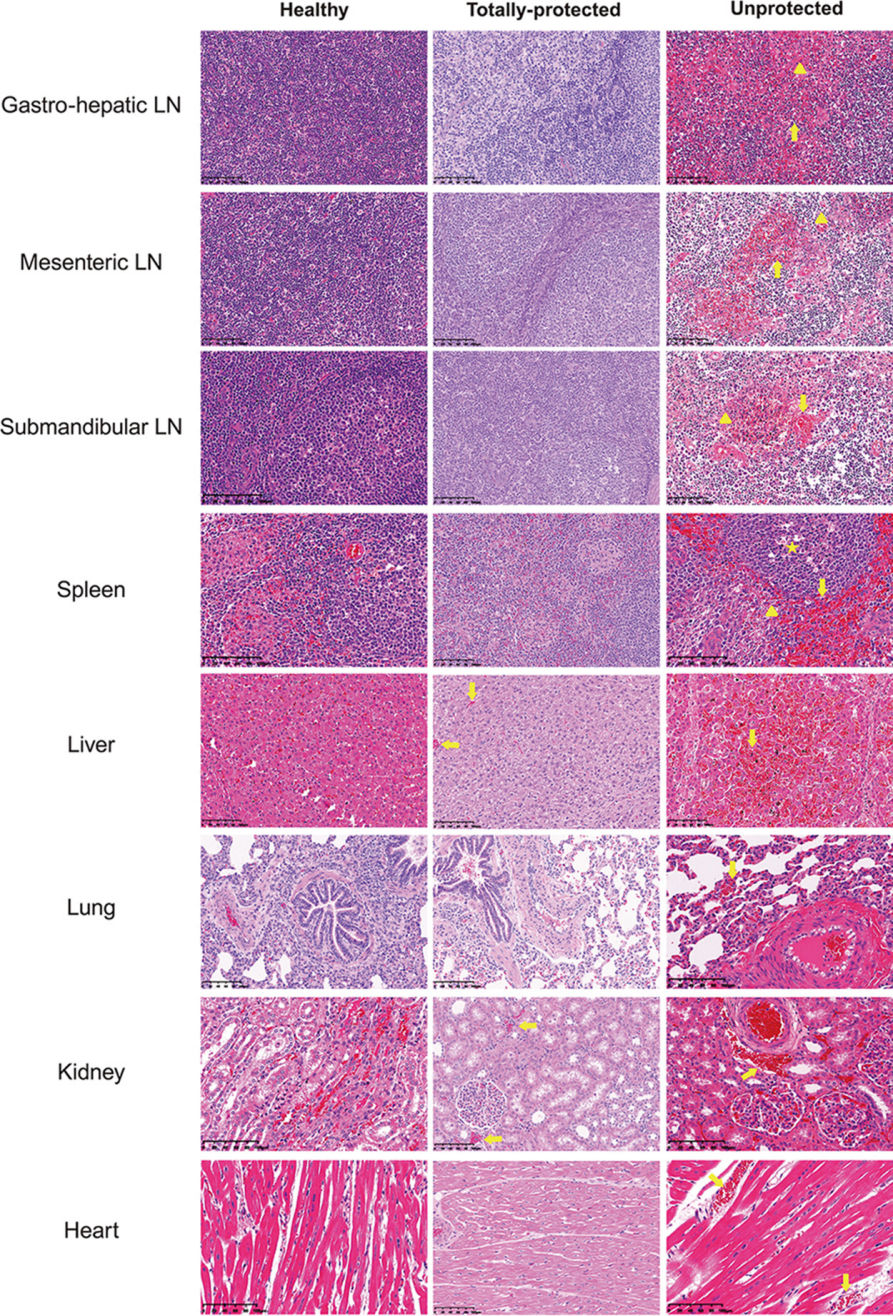
Comparison of representative microscopic lesions from healthy, unprotected and totally-protected pigs. The latter collectively came from 10^3^ HAD_50_-inoculated group. Arrow indicates severe acute and diffuse hemorrhages, and large numbers of karyorrhectic cells. Triangles indicate lymphoid depletion and loss of lymphocytes. Stars indicate infiltration of inflammatory cells.

Histological examination revealed varying autolysis in the ASFV-infected control animals and unprotected animals (Fig. 6A). As representatively demonstrated by an unprotected animal from the 10^3^ HAD_50_-inoculated group, the tissue morphology remained intact and most of tissue cells presented obvious nuclear staining. The liver, however, displayed a low staining pattern of nuclei, mainly because of karyorrhexis. More detailed observation revealed that severe hyperemia and multifocal diffuse hemorrhage were apparent in all tissue samples with the exception of the heart. Diffuse lymphoid depletion and loss of lymphocytes were most present in spleen and lymph node. Multifocal infiltration of multi-cell types, mainly neutrophils and monocytes, was predominantly viewed in liver. In contrast, paralleling the observations in one healthy animal, ASFV-related tissue lesions were significantly eased in a totally-protected animal from the 10^3^ HAD_50_-inoculated group (Fig. 6A).

### Protective efficacy is closely correlated with P30-specific antibody response.

**FIG 7:**
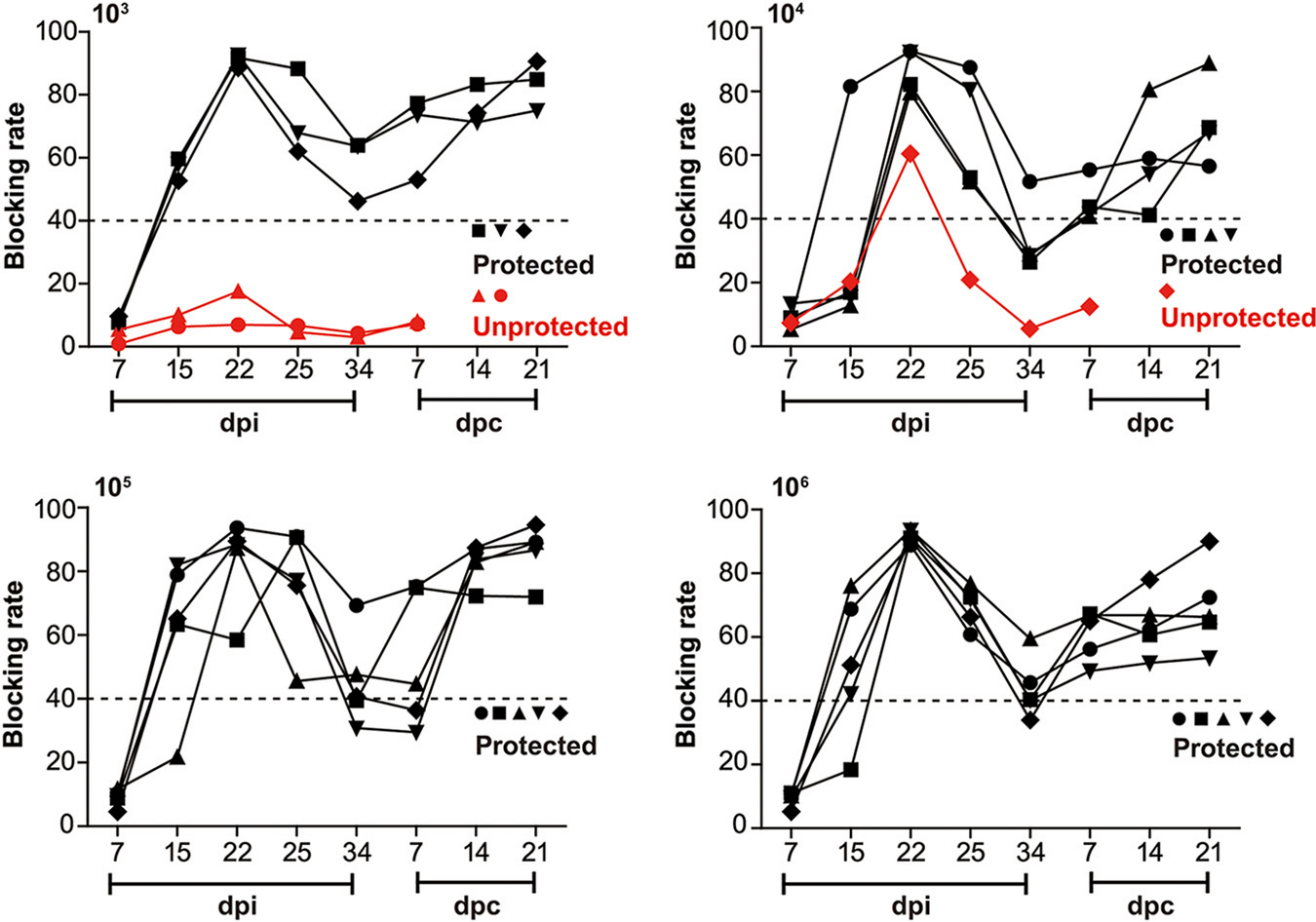
Antibody profile of experimental pigs in the period of inoculation and virulent challenge. Groups of 5 pigs inoculated with increasing titers of ASFV-Δ110-9L/505-7R (10^3^, 10^4^, 10^5^ and 10^6^ HAD_50_) were challenged i.m. with lethal wild type ASFV (10^2^ HAD_50_). Anti-ASFV P30-sepcific antibody titers was detected by ELISA. Each curve represents values from individual animals in each group.

ASFV protective humoral immunity remains poorly defined, especially the topic of effector mechanisms associated with and the viral proteins inducing antibody-mediated protective responses (27). Increasing evidence highlighted a role for ASFV neutralizing antibodies on protective immunity (23, 28). Thus, using an optimized ASFV P30-specific ELISA kit, correspondingly, the protected piglets developed a strong virus-specific p30 antibody response in the period of inoculation, with antibody titers starting to rise as early as 10 days, persisting, and peaked at 22 days after the initial infection. Significantly, but surprisingly, antibody titers began to wane over time and reached relatively low levels on the day of virulent challenge. However, with the progress of virulent challenge, continuous rebound of antibody titers was observed, with peak titers observed at the end of challenge (Fig. 7A). In comparison, unprotected pigs failed to mount a strong antibody response, with titers remaining below the antibody titer threshold (Fig. 7A), primarily suggestive of a close correlation between protective efficacy and p30 antibody response.

### Two doses are better than one.

**FIG 8:**
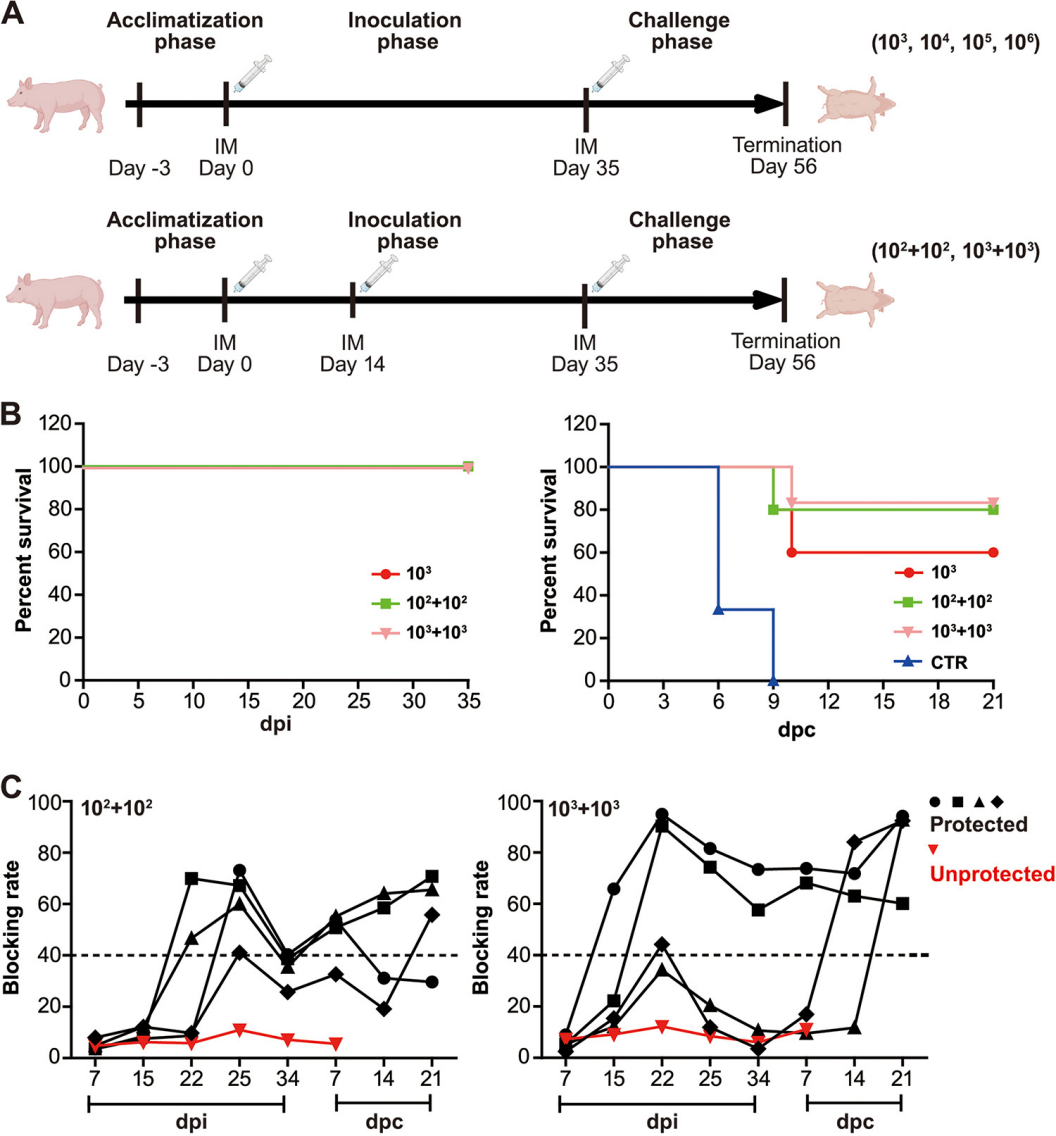
Two doses are better than one. (A) Design of animal experiments. In the one-dose inoculation procedure, after a 3-day period of acclimatization, pigs were inoculated with different doses of the ASFV-Δ110-9L/505-7R mutant at day 0. At 35 days postinoculation (dpi), pigs were challenged with 10^2^ HAD_50_ of highly pathogenic parental ASFV. After extra 21 days postchallenge (dpc), pigs had died or had been euthanized due to ethical reasons. Unlike the one-dose inoculation procedure, pigs received two doses of the mutant at 0 and 14 dpi, respectively. (B) Survival rate of pigs receiving one dose vs two doses at the period of inoculation (left panel) and challenge (right panel). (C) Antibody profile of pigs receiving one dose vs two doses in the period of inoculation and challenge.

In the next step we investigated the safety and protective efficacy of 1 dose versus 2 doses (Fig. 8A). During the inoculation period, all pigs were clinically normal, with no significant difference being observed between those received who 1 versus 2 doses (Fig. 8B; left panel). In the virulent challenge, one dose of 10^3^ HAD_50_ inoculation provided clinically significant protection in 60% pigs (3/5). As anticipated, additional protection with morbidity rates of 80% was observed in 2 doses of 10^2^ and 10^3^ HAD_50_-inoculation. Moreover, pigs receiving 2 doses demonstrated better clinical appearance, especially reduced severity and duration of fever when compared to 1 dose (Fig. 8B; right panel, and Table 4). To potentially interpret better protective efficacy imposed by 2 doses, there is evidence proving that 2 doses of 10^3^ HAD_50_ inoculation elicit noticeable strong antibody responses, with seemingly higher peak antibody titers at the end of the challenge when compared to 1 dose of 10^3^ HAD_50_ inoculation. However, 2 doses of 10^2^ HAD_50_ inoculation mounted a moderate, yet delayed, antibody response when compared to 1 dose of 10^3^ HAD_50_ inoculation, which contradicted the better protective efficacy. Those data concluded that protective immunity imposed by the mutant is mediated, and not solely by antibody immunity. Overall, 2 doses are better than 1, especially in the context of protective efficacy.

**TABLE 4:** Comparison of swine survival and rectal temperatures of pigs pre-inoculated with one dose or two doses of the mutant following infection with lethal dose of parental ASFV CN/GS/2018^*a*^

Group	Inoculation methods	Dose (HAD_50_)	No. of survivors	Time to death (mean)(days)^*b*^	Summary of fever response		
					No. of pigs with fever/total^*c*^	Duration (days)	Maximum daily temp (°C)
Control	NA^*d*^	NA	0/3	7	3/3	3.3	42.8
ASFV-Δ110-9L/505-7R	One dose	10^3^	3/5	9.5	3/5	5.5	42
	Two doses	10^2^+10^2^	4/5	9	2/5	3.5	41.6
		10^3^+10^3^	4/5	10	2/5	4	41.2

a A group of three pigs receiving a lethal dose of parental ASFV was also included.

b All animals were euthanized due to the welfare regulations according to the corresponding IACUC protocol.

c Pigs with a rectal temperature over 40°C for three consecutive days or above 40.5°C were defined as fever.

d Pigs in the control group received no ASFV-Δ110-9L/505-7R before the virulent challenge.

## DISCUSSION

ASF is notorious for high morbidity and mortality in domestic pigs and wild boars (Sus scrofa). The re-occurrence of ASF in the European Union (EU) in 2014 and first introduction of ASF to China in 2018 along with subsequent spread to neighboring Asian countries constitute a serious threat to the global pig industry. The epidemiological situation of ASF continued to deteriorate, particularly in July 2021, the disease reappeared in the Americas after maintaining ASF-free status for almost 40 years. Currently, no authorized vaccines or therapeutic options are available to control the disease, garnering significant attention in vaccination strategies in the veterinary field. Advances in live attenuated ASFV vaccine represent an effective means for preventing ASFV infection.

However, concerns about potential safety issues associated with the use of live attenuated viruses (LAVs) need to be alleviated, as recombination is one of the most frequently identified drivers of double-stranded DNA viruse evolution (29). Aligning E183L gene identified several recombination events in 16 Italian isolates and one South African isolate, inferring that these isolates have probably emerged from a common ancestor (30). Likewise, from 42 aligned ASFV genomes, there is reliable evidence proving the presence of 152 recombination events (31). Thus, it is likely that recombination facilitates ASFV combining favorable mutations to effectively generate diverse genetic strains, aiming to aid in escape from host immunity, and increase its virulence and pathogenicity (32). This property of ASFV casts a shadow on the wide use of LAVs as prevention measures, especially at the risk of reversion to wild type virulence. Previously, classically attenuated ASFVs (those created by serially passaging a highly virulent ASFV strain in cultured cells or by isolating a naturally avirulent strain) have proven to be a failure, due to conversion to lethal strains and resultant 10%–50% mortality in farmed pigs in Portugal and Spain (33, 34). These safety issues have been substantially addressed by rationally designed live viruses by applying several new approaches to viral attenuation and vaccine design, including deleterious gene mutation, altered replication fidelity, optimization of vaccination procedures, and addition of adjuvants. *In vivo* experiments demonstrated that, when serially passed in the target animals for 5 passages, ASFV HLJ/18 isolate with deletion of MGF360/505 genes progressively became more virulent and eventually resulted in death. However, the same isolate harboring deletions of CD2v and MGF360/505 genes is highly resistant to virulence conversion (34). In our study, we selectively deleted 2 interferon inhibitors located separately in different regions of the ASFV genome, which further, theoreticall, decreased the possibility of recombination and virulence conversion. However, whether the ASFV-Δ110-9L/505-7R mutant could reverse to a virulent strain during its successive replication in pigs needs more future work.

ASFV protective immunity is poorly characterized. Paralleling most viral infections, innate immunity, and both humoral and cellular immune responses appear to be crucial for protection. In this study, our results demonstrate that the protective efficacy is closely related to antibody response, proposing the notion that antibody responses weigh more in combating the virulent ASFV attack. Passively transferred ASFV antibodies are sufficient to protect pigs from lethal infection (35–37), summarizing the results that the antibody-mediated protective effect is an early event that effectively delay or prevent disease onset. However, until now, the effector mechanisms associated with, and the viral proteins inducing these antibody-mediated protective responses, are poorly defined. With the development of subunit ASF vaccines, some breakthroughs in identification of relevant ASFV protective antigens (PA), optimization of delivery/vector systems, and the breadth of their natural antigenic diversity advance the fight against the disease. The cocktails consisting of highly immunogenic ASFV structural proteins, including p30, p54, and p72, were proven to be partially effective or ineffective, even though, in most cases, robust immune responses to the PAs were mounted (38–42). Recently, a pool of 8 virally vectored ASF antigens protected 100% of pigs against the fatal disease after a challenge with a normal dose of virulent OUR T88/1 strain of ASFV (43). Opposingly, DNA-protein vaccination strategy does not protect from the challenge with the ASFV Armenia 2007 strain. Surprisingly, earlier onsets of clinical signs, viremia, and death were observed for the vaccinated animals compared to non-vaccinated pigs in the virulent challenge. Consistently, sera from immunized pigs could enhance ASFV infection *in vitro* (44). Those contradicting results point to the putative co-existence of immune enhancement mechanism and immune inhibitory mechanism that are collectively involved in ASFV pathogenesis. Based on the findings in our study, we cautiously believe that antibody response, in particular as represented by p30-specfic antibody response, has the potential to be an indicator of protection. Whether ASFV-Δ110-9L/505-7R-induced antibody response exerted bona fide antiviral activity against virulent ASFV and underlying mechanism warrants further investigation.

In this study, we described the safety, immunogenicity, and protective efficacy of a live attenuated ASFV strain harboring sequential deletions of interferon inhibitors MGF110-9L and MGF505-7R. We found that high doses of the mutant were safe and well tolerated. Meanwhile, it provided consistent sterile protection in the virulent challenge. Meanwhile, p30-specific antibody response is positively correlated with protection, potentializing the postvaccination monitoring. The sufficiency in effectiveness supports the claim that LAV strategy may be a viable vaccine option for which to fight ASF.

## MATERIALS AND METHODS

### Ethical statement.

The animal studies involving ASFV were reviewed and approved by the Ministry of Agriculture and Rural Affairs of the People’s Republic of China. For biosafety reasons, experiments were conducted in the Biosafety level 3 (BSL-3) animal facilities at Lanzhou Veterinary Research Institute (LVRI), Lanzhou, China.

### Cell cultures and viruses.

The current circulating virulent ASFV CN/GS/2018 isolate was characterized and preserved by the Regional Laboratory of ASF, Lanzhou Veterinary Research Institute. Bone marrow cell preparation was harvested from the medullary cavity of tibias obtained from 3-week-old pigs following previously described protocols (45). Bone marrow cells were cultured in growth medium supplemented with granulocyte macrophage colony-stimulating factor (GM-CSF) (10 μg/mL). After 3 days, the round and non-adherent cells started to differentiate into macrophages and adhere. The culture medium was replenished with fresh medium containing GM-CSF. Following extra 96 h of incubation, bone marrow-derived macrophages (BMDM) were gently scraped with cell scrapers and cryopreserved for further use.

### Construction and characterization of ASFV-Δ110-9L/Δ505-7R mutant.

The ASFV-Δ110-9L/505-7R mutant was constructed from a highly virulent genotype II isolate by 2 successive homologous-recombination procedures. Firstly, MGF505-7R gene encompassing a 1584-bp region was replaced with a 917-bp cassette containing the reporter gene cassette p72eGFP. The resulting recombinant ASFV-Δ505-7R mutant was purified by using a series of limited dilution on monolayers of BMDM cultures and applied to the second recombinant event. Analogously, a p72mCherry reporter cassette of 908 bp in size was engineered into a 873-bp region of MGF110-9L gene, resulting in a two-gene-deleted recombinant ASFV-Δ110-9L/505-7R mutant expressing eGFP/mCherry signals.

Molecular diagnosis of ASFV-Δ110-9L/505-7R mutant was performed by PCR with 2 primer pairs targeting outside or inside of the deleted gene, referred to as flanking primer pairs and centering primer pairs, respectively. In detail, viral DNA was extracted from virus suspension and subjected to PCR amplification. PCR products were detected by agarose gel electrophoresis and subjected to sanger sequencing. The detailed sequences of primer pairs are provided in Table S1.

To further assess the emergence of unwanted genetic changes outside the deleted MGF110-9L and MGF505-7R, NGS of the mutant was performed as previously described. In detail, BMDM cells were infected with ASFV-Δ110-9L/505-7R at a multiplicity of infection (MOI) of 0.1. Following 72 h of incubation, the cell culture supernatant was harvested, pooled, clarified, and ultracentrifuged. ASFV DNA was extracted using the SDS method, and harvested DNA was sent to Allwegene Technology Co., Ltd. (Beijing) for further analysis.

### Assessment of growth kinetics.

The replication kinetics of parental ASFV and ASFV-Δ110-9L/505-7R mutant were assessed in the BMDM cultures. At 24 h before experimentation, BMDM cells were seeded at a concentration of 5 × 10^5^ cells/well in a 12-well plate. The next day, monolayers were infected with ASFV at an MOI of 0.1 and the inoculum was replaced with growth medium containing GM-CSF (10 μg/mL) after 2 h of incubation. At the indicated time points, the whole cell culture was frozen and thawed, clarified and titrated by the HAD_50_ assay, according to the Spearman-Karber method as outlined in the Manual of Diagnostic Tests and Vaccines for Terrestrial Animals (World Organisation for Animal Health [OIE, 2014]). The plates were observed for hemadsorption over a period of 6 days.

### The triplex real-time rPCR assay.

Triplex real-time PCR (rPCR) primers and probes were designed according to the sequence of ASFV CN/GS/2018 by using Primer Express 3.0 software (Applied Biosystems, Foster City). Three pairs of primers and probes targeting the conserved sequence region of the B646L gene (encoding p72 protein), MGF110-9L and MGF505-7R, respectively, were designed and applied in the following experiment. For multiplexing, the probe for the B646L gene was labeled with the 5′-reported dye Texas Red-X and the 3′-quencher BHQ2, the probe for MGF110-9L was labeled with the 5′-reported dye 6-Carboxyfluorescein (FAM) and the 3′-quencher BHQ1, and the probe for MGF505-7R was labeled with the 5′-reported dye Cy5 and the 3′-quencher BHQ2. All the primers and probes were synthesized by GenScript. The specificity and sensitivity of the assay were examined by our lab and data were not shown. All the sequences will be shared upon request.

### Assessing the stability of genetic modifications in the mutant by repeated passages in BMDM.

The mutant was repetitively propagated in BMDM cell cultures using 2 different passage strategies. BMDM cells (5 × 10^6^ cells/well) were dispersed in 6-well cell culture plates and incubated for 2 days. The cells were then inoculated with the mutant at an MOI of 0.1. After 2 h of incubation at 37°C, following repeated washing steps, and incubated for another 72 h. In passage scheme 1, cells simultaneously positive for eGFP/mCherry signals were isolated by Flow cytometry sorting and repeatedly frozen and thawed. The virus suspension (P1) was titrated and inoculated into fresh BMDM at 0.1 MOI for a second round of culture to harbor P2. In passage scheme 2, whole cell cultures were repeatedly frozen and thawed. After clarification, virus suspension (P1) was titrated and inoculated into fresh BMDM at 0.1 MOI for another 72 h to harbor P2. A total of 30 successive passages were performed and virus suspensions were stored at −80°C until use. To assess the stability of genetic deletions in the mutant, virus suspensions from passages at 5 (P5), 10 (P10), 20 (P20), and 30 (P30) were subjected to triplex rPCR assay.

### Animal experiments.

All animals were handled in strict accordance with good animal practice according to the Animal Ethics Procedures and Guidelines of the People’s Republic of China, and the study was approved by the Animal Ethics Committee of Lanzhou Veterinary Research Institute (LVRI), Chinese Academy of Agricultural Sciences (CAAS), Lanzhou, China.

The 4- to 5-week-old Duroc-Landrace-Yorkshire piglets (male/female) with body weight of 15–18 kg were obtained from a local pig farm with high biosecurity standards and hygiene. Diagnostic tests confirmed the absence of ASFV-specific P30 antibodies and the clinically common porcine viruses. Following a 3-day period of acclimatization, piglets were randomly divided into groups of 5 pigs with each group housed in an isolated room. In the one-dose inoculation method, pigs were inoculated IM with increasing doses of ASFV-Δ110-9L/505-7R mutant, ranging from 10^3^ HAD_50_ to 10^6^ HAD_50_ (day 0). At 35 days postinoculation (dpi), pigs were exposed to a virulent challenge with 10^2^ HAD_50_ of parental ASFV. At extra 21 days postchallenge (dpc), pigs had died or had been euthanized using pentobarbital at the humane endpoint. Further, the control group of 3 pigs was directly administered with 10^2^ HAD_50_ of parental ASFV.

In the two-dose inoculation experiments, 2 groups of pigs were inoculated IM, either with 10^2^ or 10^3^ HAD_50_ of ASFV-Δ110-9L/505-7R. After 2 weeks, each pig received the same dose of the mutant. Following a 35 day observational period, pigs were exposed to a virulent challenge the same as the previous one-dose inoculation experiment.

Clinical signs, including rectal temperatures, were recorded daily during the entire experiment. Survival and time-to-death were recorded as previously described (46). To assess viremia and immune response, blood samples (blood with EDTA and serum) were collected from the jugular vein at 7, 15, 22, 25, and 34 dpi and at 7, 14, and 21 dpc. To assess the extent of virus shedding, oral, rectal, and nasal swabs were obtained at the same time points as blood samples. Necropsy was carried out on unprotected and totally-protected pigs from 10^3^ HAD_50_-inoculated group, and tissue samples were simultaneously collected either by snap-freezing for viral detection or by fixation in 10% formalin for histology and immunohistochemistry.

### ASFV DNA load detection.

In the laboratory, the swabs were directly dipped in 500 μL of 1X phosphate buffered saline (PBS) and subjected to 3–4 freeze-thaw cycles, followed by repetitive high-speed centrifugation. The supernatants were directly subjected to detection of ASFV genomic DNA through qPCR method. In detail, we used TaqMan quantitative PCR assay targeting P72 gene for ASFV copy number quantification by using the primers and probe in Table S1. The assay was performed using the Pro *Taq* HS Premix Probe qPCR kit (TaKaRa, AG11704). Each 25 μL reaction consisted of 12.5 μL 2× mix buffer, 3 μL DNA template, 6.5 μL H_2_O and 3 μL primers/probe. The qPCR was performed (applied biosystems QuantStudio5) as followings: enzyme activation at 95°C for 2 min (1 cycle), followed by denature at 95°C for 7 s and anneal at 60°C for 12 s (3 cycle). Finally, we denatured at 95°C for 6 s and annealed at 58°C for 11 s (40 cycle). In the case of blood and tissues, viral DNA was extracted using a Tissue DNA Kit (OMEGA, D3396-02) according to the manufacturer’s instructions. Viral DNA was determined by absolute quantification using the TaqMan-based real-time PCR assay targeting the VP72 DNA sequence. The results were calculated based on a standard curve depicting average cycle of quantification (Ct) values plotted against the number of target copy number per reaction, and expressed in absolute terms of DNA equivalents per mililiter (genome copies/mL).

### P30-specific antibody detection.

Detection of ASFV P30-specific antibodies in the serum was performed using our optimized ELISA kit (Lanzhou Shouyan Biotechnology Co., Ltd) (Lot number: glwe-sj). All the experiments were performed strictly according to the instructions supplied by the manufacturers. Assays were read on an enzyme-labeled instrument and calculated based on the standard curve constructed independently in each assay.
