# Supplementary material for: Sequential Deletions of Interferon Inhibitors MGF110-9L and MGF505-7R Result in Sterile Immunity against the Eurasia Strain of Africa Swine Fever
Source: J Virol. 2022 Oct 5;96(20):e01192-22. doi: 10.1128/jvi.01192-22 (PMC9599437; doi:10.1128/jvi.01192-22)
Supplement: Supplemental file 1 — Table S1 and Fig. S1 to S5. Download jvi.01192-22-s0001.pdf, PDF file, 3.1 MB [file jvi.01192-22-s0001.pdf]

Supplementary files to

Sequential deletions of interferon inhibitors MGF110-9L and MGF505-7R result in sterile immunity against the Eurasia strain of African swine fever

Supplementary Table 1 List of primers and primer sequences.

Primer			Sequence
**MGF110-9L**	Centering primers	Forward	5’-TTACAGGATATGTCAGCGAC-3’
		Reverse	5’-GCAAATAAAGGAGGATAGGGTC-3’
	Flanking primers	Forward	5’-TGGTCGTAAAGGATTCGGTGG-3’
		Reverse	5’-GCCCGCTATTTTGACCGTTG-3’
**MGF505-7R**	Centering primers	Forward	5’-ACTGGCATGTTCTCCTCCCTT-3’
		Reverse	5’-ATTCTTTCCGCGGGATTTTCC-3’
	Flanking primers	Forward	5’-AGTGGAAGGCGATTTTTGGTAG-3’
		Reverse	5’-TGCTTGCCTAAGGGCTCAAG-3’
**P72 (traditional PCR)**		Forward	5’-ATATTGCGTCTACTGGGGCG-3’
		Reverse	5’-AGTTCGGATGTCACAACGCT-3’
**P72 (Taqman qPCR)**		Forward	5’-ATGGAAATTCCCTAGACGAA-3’
		Reverse	5’-CACTGGTTCCCTCCACCGAT-3’
		TaqMan probe	5’-FAM-ACCTCCTGGCCAACCAAGTGCT-BHQ1-3’

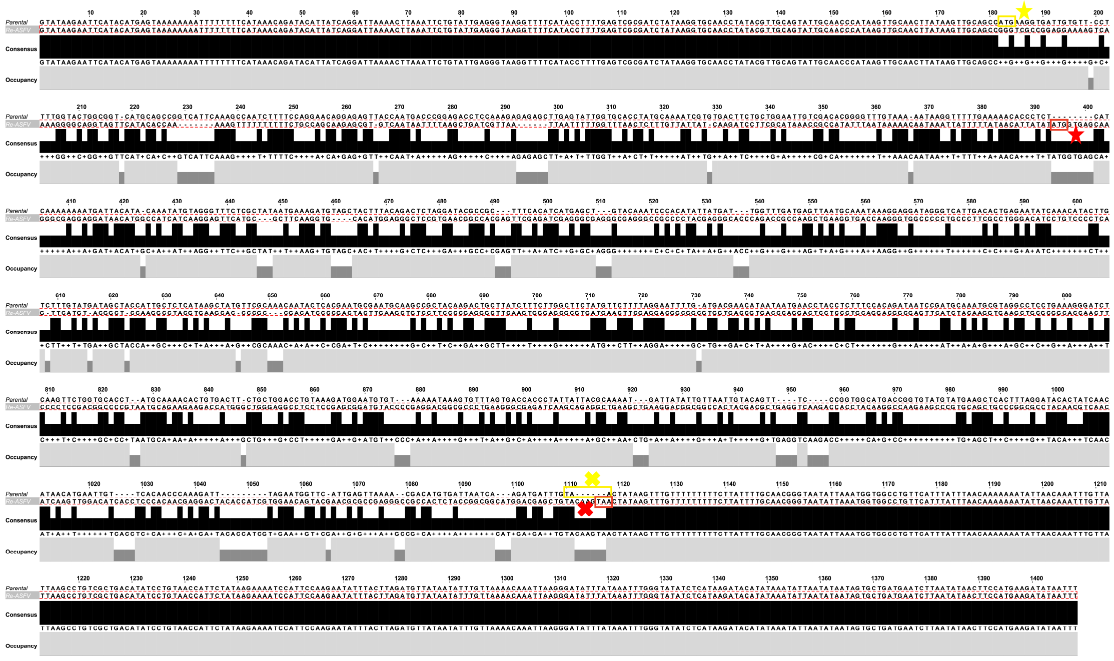

sFig. 1 Sanger sequencing validation of the precision of genetic modifications in the position of MGF110-9L gene in the ASFV-ΔMGF110-9L/505-7R mutant. The PCR-amplified fragments with the genomic DNA from parental ASFV or ASFV-ΔMGF110-9L/505-7R mutant were subjected to sanger sequencing. Pairwise sequence alignment demonstrated that MGF110-9L ORF was precisely replaced by p72-mCherry reporter cassette in the mutant. Yellow arrow and cross indicated the start codon and stop codon of MGF110-9L ORF in the parental ASFV, while red arrow and cross indicated the start codon and stop codon of mCherry ORF in the mutant.

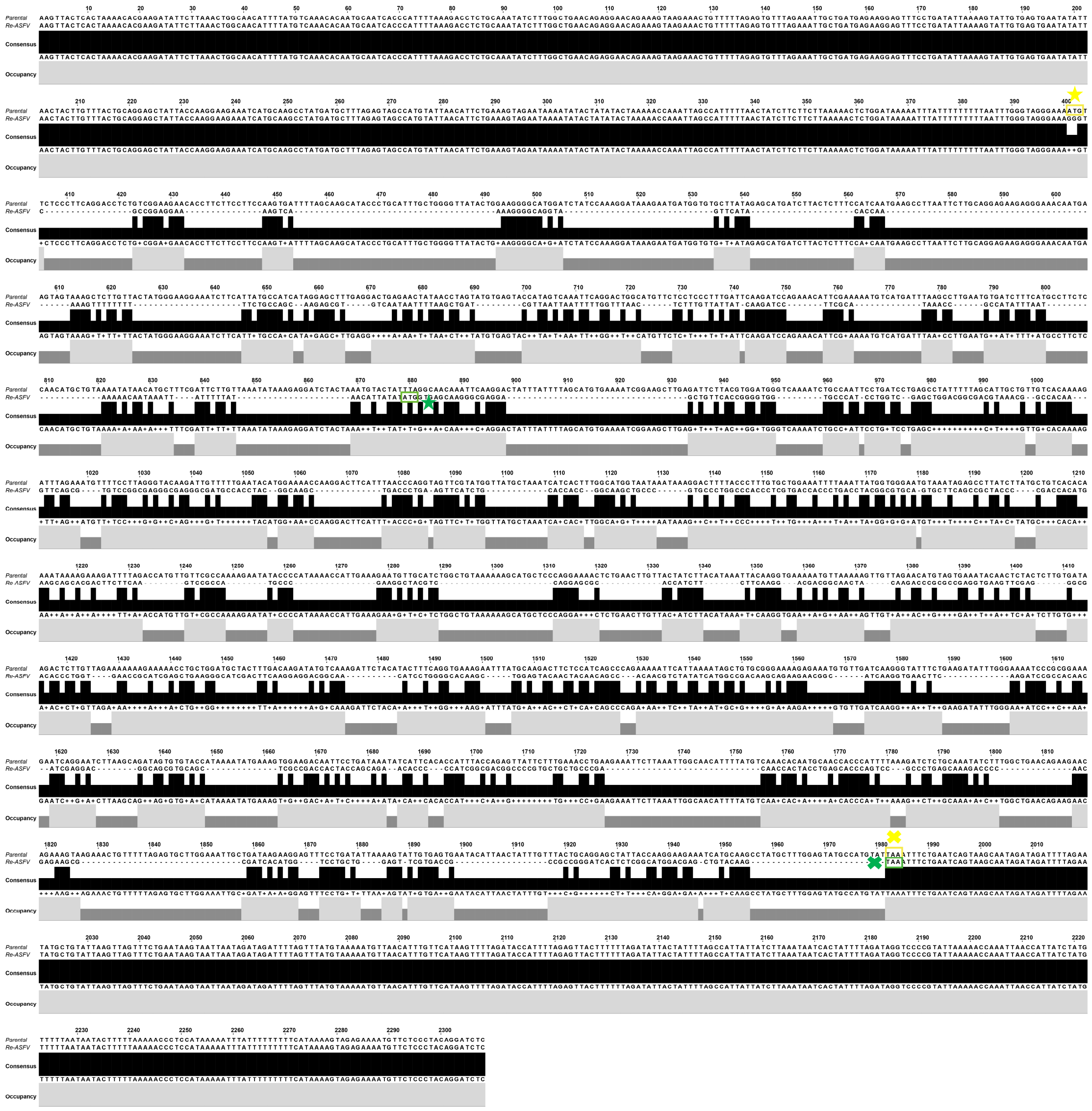

sFig. 2 Sanger sequencing validation of the precision of genetic modifications in the position of MGF505-7R gene in the ASFV-ΔMGF110-9L/505-7R mutant. The PCR-amplified fragments with the genomic DNA from parental ASFV or ASFV-ΔMGF110-9L/505-7R mutant were subjected to sanger sequencing. Pairwise sequence alignment demonstrated that MGF505-7R ORF was precisely replaced by p72-eGFP reporter cassette in the mutant. Yellow arrow and cross indicated the start codon and stop codon of MGF110-9L ORF in the parental ASFV, while green arrow and cross

indicated the start codon and stop codon of eGFP ORF in the mutant.

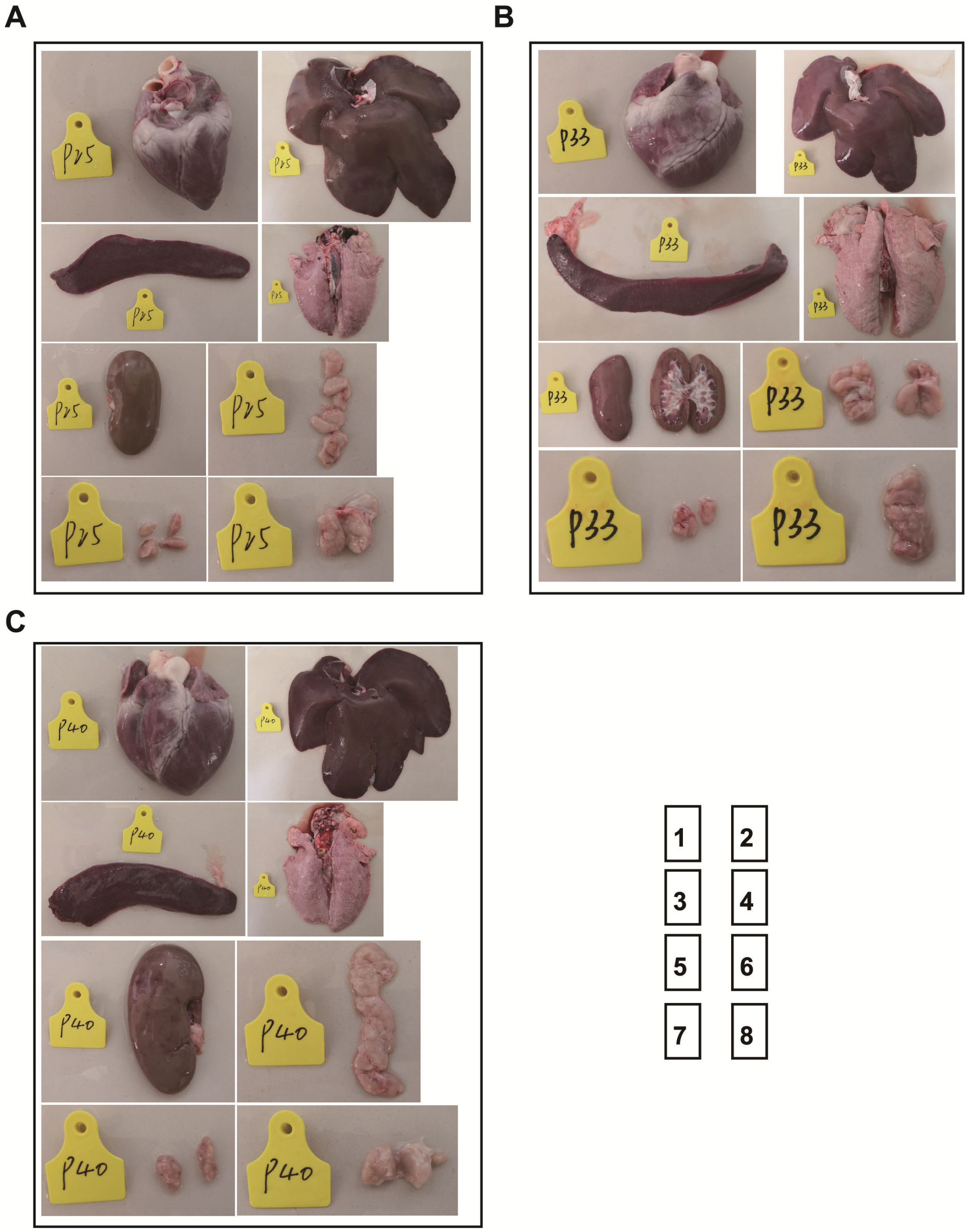

sFig. 3 Postmortem lesion obtained from totally-protected pigs in the 10^4 HAD_{50} group (A), 10^5 HAD_{50} group and 10^6 HAD_{50} group on ① heart, ② liver, ③ spleen, ④ lung, ⑤ kidney, ⑥ submandibular lymph node, ⑦ gasto-hepatic lymph node and ⑧mesenteric lymph node.

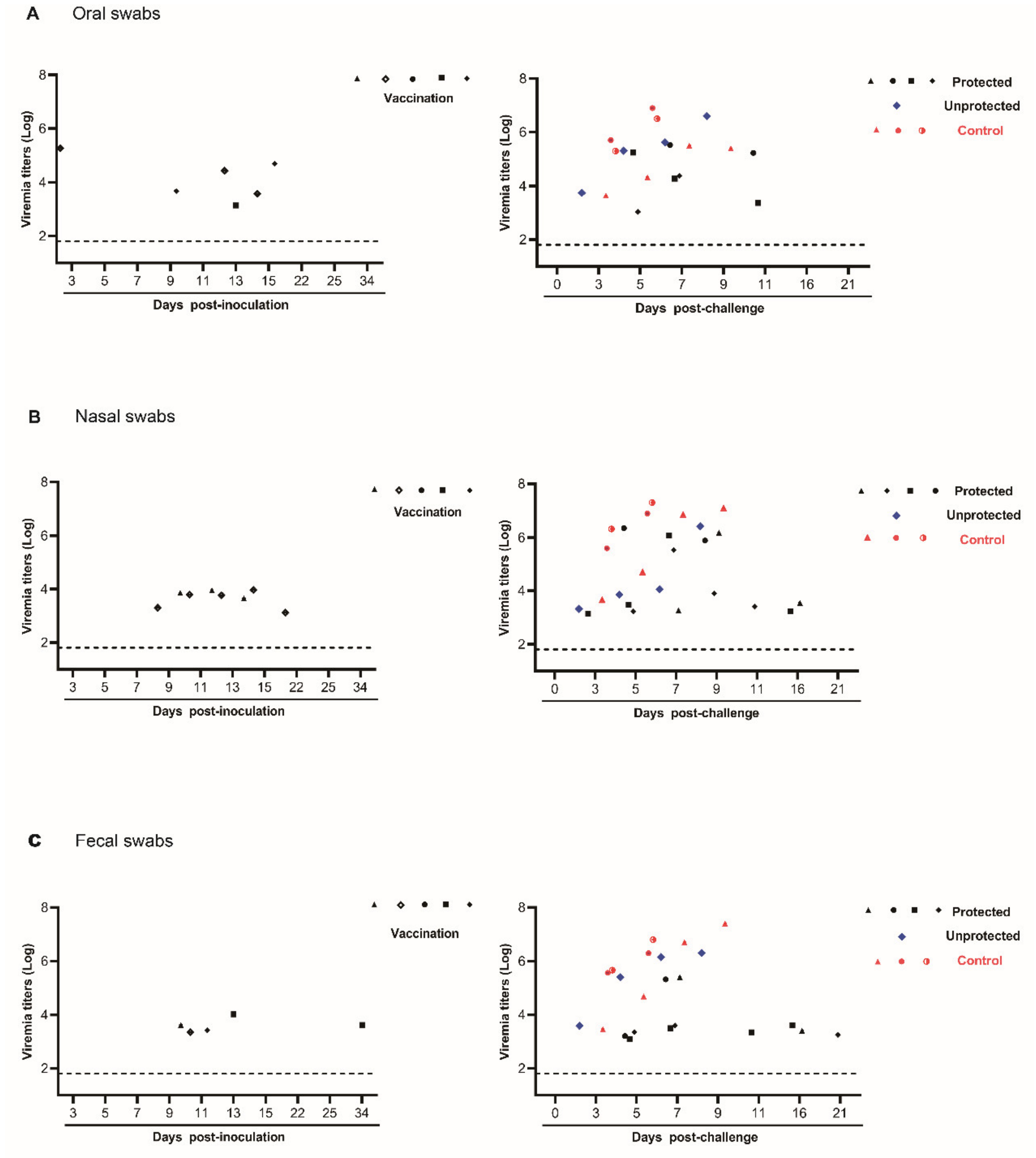

sFig. 4 Virus sheddings in fecal and oro-nasal samples collected from 10^4-inoculated pigs. The dashed line indicates the lower limit of detection.

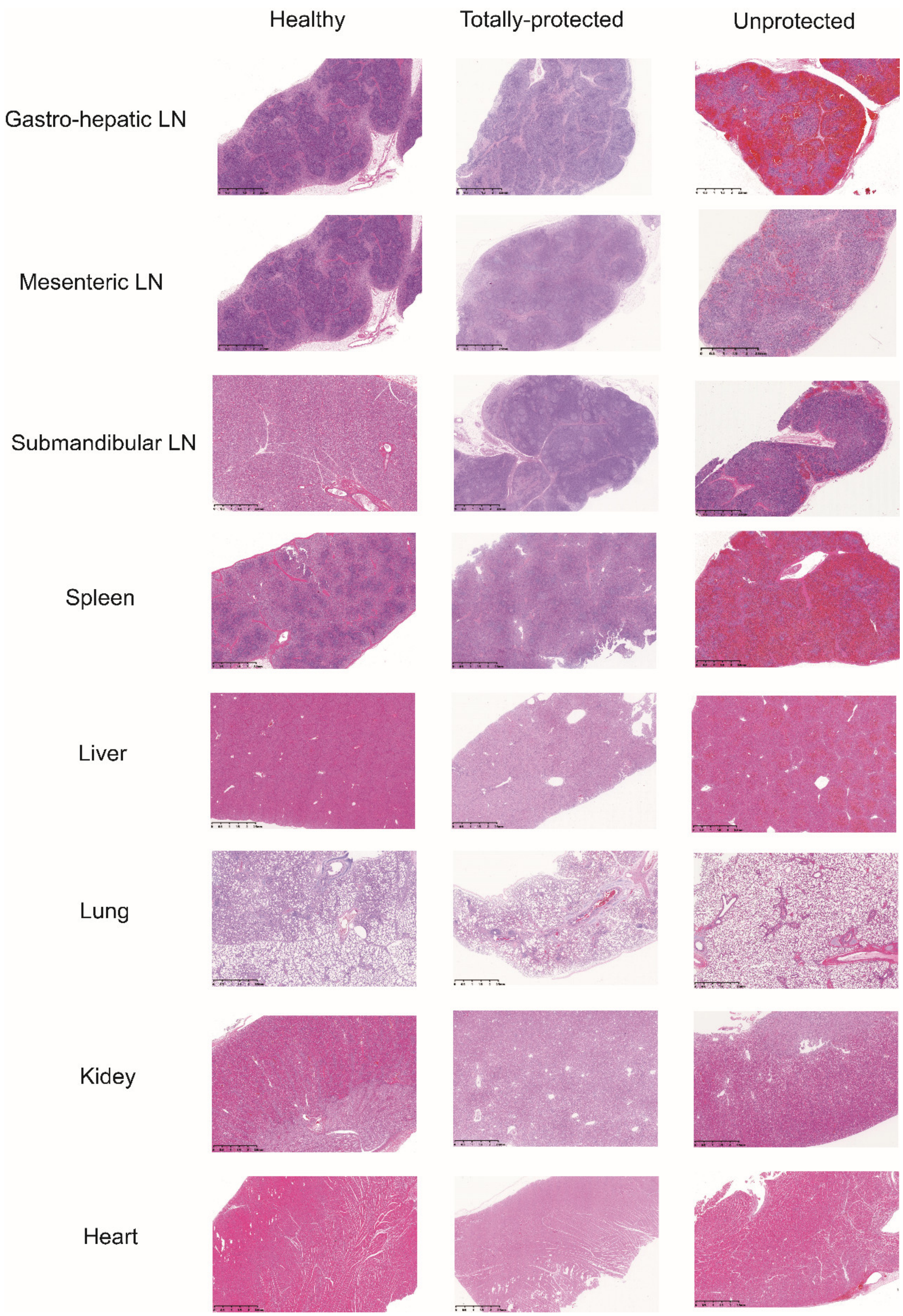

sFig. 5 Comparison of representative microscopic lesions from healthy, unprotected and totally-protected pigs with a low magnification.
